# Valuing increased zinc (Zn) fertiliser-use in Pakistan

**DOI:** 10.1007/s11104-016-2961-7

**Published:** 2016-07-08

**Authors:** Edward J. M. Joy, Waqar Ahmad, Munir H. Zia, Diriba B. Kumssa, Scott D. Young, E. Louise Ander, Michael J. Watts, Alexander J. Stein, Martin R. Broadley

**Affiliations:** 1 Faculty of Epidemiology and Population Health, London School of Hygiene & Tropical Medicine, Keppel St, London, WC1E 7HT UK; 2 Food and Agriculture Organization of the United Nations, NARC Premises, Park Road, Islamabad, Pakistan; 3 Research & Development Section, Fauji Fertilizer Company Ltd, Rawalpindi, Pakistan; 4 School of Biosciences, University of Nottingham, Sutton Bonington Campus, Loughborough, LE12 5RD UK; 5 Inorganic Geochemistry, Centre for Environmental Geochemistry, British Geological Survey, Keyworth, Nottingham, NG12 5GG UK; 6 Agricultural Economist, Brussels, Belgium

**Keywords:** Agronomic biofortification, Benefit cost ratio (BCR), Cost-benefit analysis, Disability adjusted life year (DALY), Fertiliser subsidy, Food security, *Triticum aestivum* L, Wheat

## Abstract

**Background and aims:**

Use of zinc (Zn) fertilisers may be cost-effective in increasing crop yields and in alleviating dietary Zn deficiency. However, Zn fertilisers are underutilised in many countries despite the widespread occurrence of Zn-deficient soils. Here, increased Zn fertiliser-use scenarios were simulated for wheat production in Punjab and Sindh Provinces, Pakistan. Inputs and outputs were valued in terms of both potential yield gains as well as health gains in the population.

**Methods:**

The current dietary Zn deficiency risk of 23.9 % in Pakistan was based on food supply and wheat grain surveys. “Disability-adjusted life years (DALYs) lost” are a common metric of disease burden; an estimated 245,000 DALYs y^−1^ are lost in Punjab and Sindh due to Zn deficiency. Baseline Zn fertiliser-use of 7.3 kt y^−1^ ZnSO_4_.H2O was obtained from published and industry sources. The wheat area currently receiving Zn fertilisers, and grain yield responses of 8 and 14 % in Punjab and Sindh, respectively, were based on a recent survey of >2500 farmers. Increased grain Zn concentrations under Zn fertilisation were estimated from literature data and converted to improved Zn intake in humans and ultimately a reduction in DALYs lost.

**Results:**

Application of Zn fertilisers to the area currently under wheat production in Punjab and Sindh, at current soil: foliar usage ratios, could increase dietary Zn supply from ~12.6 to 14.6 mg *capita* ^−1^ d^−1^, and almost halve the prevalence of Zn deficiency, assuming no other changes to food consumption. Gross wheat yield could increase by 2.0 and 0.6 Mt. grain y^−1^ in Punjab and Sindh, respectively, representing an additional return of US$ >800 M and an annual increased grain supply of 19 kg *capita* ^−1^.

**Conclusions:**

There are potential market- and subsidy-based incentives to increase Zn fertiliser-use in Pakistan. Benefit-Cost Ratios (BCRs) for yield alone are 13.3 and 17.5 for Punjab and Sindh, respectively. If each DALY is monetised at one to three fold Gross National Income per capita on purchasing power parity (GNI_PPP_), full adoption of Zn fertiliser for wheat provides an additional annual return of 405–1216 M International Dollars (I$) in Punjab alone, at a cost *per* DALY saved of I$ 461–619.

**Electronic supplementary material:**

The online version of this article (doi:10.1007/s11104-016-2961-7) contains supplementary material, which is available to authorized users.

## Introduction

Zinc (Zn) is an essential nutrient for all organisms, with potential roles in 1000s of proteins in plants and humans (Broadley et al. 2007). Crops respond to Zn fertilisers on many soil types. For example, increases in wheat yield and production at a national scale have been reported in Turkey following the adoption of Zn fertilisers (Cakmak 2008; Cakmak et al. 2010). However, Zn fertilisers remain little utilised globally, and approximately half of all soils used for cereal production are likely to be Zn deficient (Cakmak et al. 1999; Alloway 2008; Ahmad et al. 2012). These soils include widespread areas of the Indo-Gangetic Plains in South Asia, where intensive rice-wheat cropping systems are practiced (e.g. Nayyar et al. 2001). For example, soils of the Indus Plains of Pakistan are mostly derived from calcareous parent material from the Himalayas, which is deposited as alluvial material by the Indus River and its tributaries, or as loess deposits in the northern parts of the Indus Plains (FAO 1973). These calcareous soils support the majority of crop production in Pakistan, which covers 21.4 Mha (PBS 2009). These soils generally have low organic matter (0.4–0.7 %) and free calcium carbonate (CaCO_3_), which buffers the pH within the range of 7.5–8.4 with 100 % base saturation, and have a cation exchange capacity (CEC) dominated by Ca. These factors tend to restrict the phyto-availability of Zn and other elements such as boron (B), thereby limiting crop yields in the absence of their fertilisers.

Many field studies have reported crop yield increases in response to Zn fertilisers. Using these studies, it is straightforward to monetise Benefit-Cost-Ratios (BCRs) based on increased crop output *per* additional input of Zn. For example, in a review of field studies in Pakistan, an application of 5 kg ha^−1^ (i.e. ZnSO_4_. H2O equivalents, containing ~33 % Zn) increased grain yields of wheat by >10 %, at a mean BCR of 7:1 (range 1.3–11:1; NFDC 1998). In addition to yield increases in the year of Zn fertiliser application, there may be beneficial residual effects of Zn fertilisers for subsequent crops for three or more years (NFDC 1998; Singh and Shivay 2013; Manzeke et al. 2014; Wang et al. 2015a). However, despite these potential financial returns, Zn fertilisers remain little utilised in Pakistan and elsewhere, for several reasons. These include a lack of quality product availability/access and farmer-awareness. There is also evidence of an unwillingness to pay for Zn fertilisers due to mistrust of product quality and labelling (NFDC 1998). The effects of subsidies that focus primarily on the supply of macronutrient fertilisers, and a lack of farmer-access to longer-term credit, can also discourage longer-term soil fertility-building and lead to imbalanced fertiliser-use at a farm level (Khan et al. 2010). Furthermore, there are scientific knowledge gaps in terms of deploying balanced fertiliser applications in soils with multiple macro- and micro-nutrient stresses including B-deficiency and saline/sodic soil systems, which are prevalent in Pakistan (Ahmad and Muhammad 1998).

The use of Zn fertilisers can also increase Zn concentration in the endosperm of cereal-grains, thereby reducing risks to consumers of dietary Zn deficiency (Cakmak 2009; White and Broadley 2009, 2011; Cakmak et al. 2010; Joy et al. 2015b). The impact of increased dietary Zn intake and subsequent reductions in Zn deficiency within populations can be quantified using a Disability-adjusted life years (DALYs) framework (Murray 1994; Stein et al. 2005, 2006; Stein 2014). The use of DALYs allows health losses due to morbidity, injury and mortality to be expressed in a single metric. This measure can then also be used to quantify the impact of public health interventions in terms of “DALYs saved” and to rank potential interventions by taking their underlying costs into account. In this case the key metric is the “cost *per* DALY saved” for each intervention with a lower cost *per* DALY representing a more cost-effective intervention.

For example, Joy et al. (2015b) estimated that foliar Zn application to 75 % of cereals in 10 countries in sub-Saharan Africa could increase Zn intakes by ~1.0 mg Zn *capita* ^−1^ d^−1^, saving 0.5 M DALYs annually at a cost of US$ 46–347 *per* DALY saved. In comparison, Stein et al. (2006) estimated that biofortification of high-Zn rice and wheat varieties through breeding could save up to 55 % of the 2.8 M DALYs lost annually due to Zn deficiency in India at a cost of US$ 0.68–8.80 *per* DALY saved. Fiedler et al. (2013) estimated that fortifying maize meal with a premix containing Zn at large-scale mills in Zambia could save 5657 DALYs annually, of which 1757 were due to Zn deficiency, at a cost of US$ 401 *per* DALY saved. While the cost-effectiveness of these interventions varies considerably, it is also necessary to take into account the socio-economic realities in each target country. The Commission on Macroeconomics and Health of the World Health Organization (WHO 2001) suggested valuing each DALY lost at the national *per capita* income (or even at three times the *per capita* income). Under this criterion the above (bio) fortification interventions are likely to be worth implementing in their respective settings.

The aim of this study was to compare the potential financial return from Zn fertilisers, both in terms of yield and public health, using yield BCRs and DALYs. Combining yield and health-based valuations of an increased supply of micronutrients that is delivered through fertilisers has not previously been attempted to our knowledge. Pakistan was chosen as a case study because: (1) of a high prevalence of Zn deficient soils and human dietary Zn deficiency; (2) a large proportion of dietary energy intake is from wheat; (3) of good data availability on fertiliser-use from public and private sector sources, notably the National Fertilizer Development Centre (NFDC). The NFDC was set up by the Government of Pakistan in 1977, with support from United Nations Development Programme (UNDP), the Food and Agriculture Organization (FAO) of the UN, the Governments of Norway, the Netherlands and other international donors (http://www.nfdc.gov.pk/about.html). The NFDC compile fertiliser-use statistics at a district-level scale that are used for policy support and to guide R&D and communication priorities.

## Methods

Baseline assumptions for yield and health-based valuations were made using recent data from Pakistan for: (1) dietary Zn supply from wheat, grown with and without Zn fertilisers; (2) current Zn fertiliser practices and costs; (3) wheat production statistics and support prices; (4) estimated disease burden in terms of lost DALYs due to Zn deficiency. Data were obtained from published and unpublished sources, including a large-scale survey of farmer practices in Punjab and Sindh. The value of increasing Zn fertiliser-use from this baseline was estimated using scenario changes combined with explicit assumptions described below. The study focuses on Punjab and Sindh as the major agricultural production regions of Pakistan and because of data availability. The study focuses on wheat as the dominant dietary source of energy and Zn in Pakistan (FAO 2015).

### Dietary Zn supply from wheat under baseline and Zn-fertilised scenarios

**Fig. 1:**
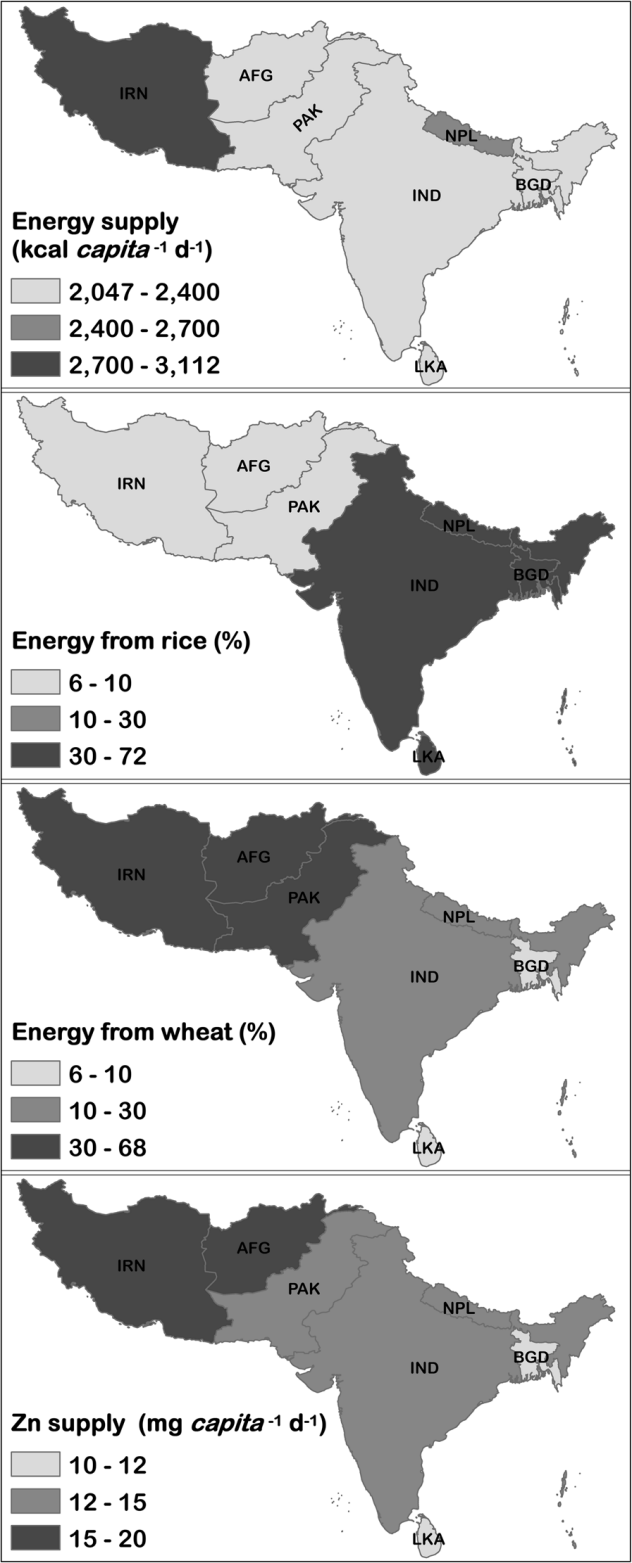
Dietary energy supply in 2010 for S. Asia, **a** from all foods; **b** from rice; **c** from wheat. **d** dietary Zn supply. Data are from Kumssa et al. (2015b)

Recent published estimates of dietary energy supply from wheat, rice and other main food sources were obtained from Kumssa et al. (2015b). The supply of Zn for Pakistan was estimated to be 13.9 mg Zn *capita* ^−1^ d^−1^ in 2010 (Kumssa et al. 2015b; Fig. 1). This is based on FAO Food Balance Sheets for 2010 (FAO 2015), which report a supply of 311 g *capita* ^−1^ d^−1^ of wheat grain and USDA food composition data of 29.1 mg Zn kg^−1^ (USDA 2013)*.* Wheat would therefore supply 8.3 mg Zn *capita* ^−1^ d^−1^ after correcting for edible portion (Supplementary Table 1). Wheat is the major staple food in Pakistan, contributing ~37 % of the daily energy in the Pakistan food supply system. The supply of rice is much less at 37 g *capita* d^−1^ (Fig. 1).

USDA data is likely to overestimate the Zn concentration of wheat grain grown on soils with low-Zn availability such as in Pakistan. Thus, we adjusted estimates of dietary Zn supply, using data from grain samples of wheat grown in Pakistan on soils of contrasting Zn status. Collection of these grain samples was described previously (Zia et al. 2015). The mean/median grain Zn concentration was 24.9/24.5 mg kg^−1^ (standard deviation = 4.8; range = 15.1–39.7, *n* = 75; Munir H. Zia and Michael J. Watts, unpublished observations). Using a mean grain Zn concentration value of 24.9 mg kg^−1^ represents a supply of 7.1 mg Zn *capita* ^−1^ d^−1^ from wheat for Pakistan in 2010, after correcting for edible portion, and a baseline dietary Zn deficiency risk of 23.9 % compared to 16.1 % based on USDA wheat composition data. Both estimates assume a national population-weighted Estimated Average Requirement (EAR) of 10.4 mg Zn *capita* ^−1^ d^−1^ (Kumssa et al. 2015b). These baseline estimates of dietary Zn deficiency are likely to underestimate the extent of nutritional Zn deficiency. For example, the 2011 Pakistan National Nutrition Survey Report (NNS 2011) reported that 42 % (*n* = 5953) and 48 % (*n* = 791) of non-pregnant and pregnant women respectively and 37 % (*n* = 2499) of children <5 years were Zn-deficient, based on having plasma Zn concentrations <60 μg Zn dL^−1^. This discrepancy is likely to be due to: (1) an over-estimate of dietary Zn supply, considering the use of USDA food composition data for food items other than wheat and considering that food supply data does not account for household-level wastage; (2) the assumption of a 25 % coefficient of variation in dietary Zn intakes may underestimate the inequality of access to food due to socio-economic or social exclusion factors (Joy et al. 2014, 2015a, b; Kumssa et al. 2015a, b; Kuper et al. 2015); and (3) nutritional Zn deficiency can occur despite adequate dietary Zn supply due to factors such as diarrhoeal disease.

### Zn fertiliser practices and costs

Fertiliser-use is currently unbalanced in Pakistan. In 2012/13, total fertiliser availability was 3865 kilotonnes (kt; NFDC 2013). This comprised 2975 kt of N, 863 kt of phosphate (P_2_O_5_), and 27 kt of potash (K_2_O). The offtake (consumption, nutrient weight) of fertilizer during this period was 3621 kt, comprising 2853 kt N, 747 kt phosphate, and 21 kt potash. The difference between availability and offtake is the amount carried forward into the next fiscal year, i.e. 2013/14. Urea (CO(NH_2_)_2_; 46:0:0 NPK) comprised 84 % of total N fertilisers used in 2012/13 (NFDC 2013). In terms of nutrient weights, 2391 kt of N use was from urea, 112 kt was from calcium ammonium nitrate (CAN; 5Ca(NO_3_)_2_.NH_4_NO_3_.10H_2_O; ~26:0:0), 242 kt was from diammonium phosphate (DAP; (NH_4_)_2_HPO_4_; 18:46:0), 103 kt was from nitrophos (NP; 22:20:0), with much smaller amounts of N from NPK fertilisers of varying composition and <1 kt from monoammonium phosphate (MAP; NH_4_H_2_PO_4_; 11:52:0). The most widely used form of phosphate in Pakistan is DAP (NFDC 2013). In 2012/13, this comprised 83 % of total phosphate fertiliser-use. In terms of P_2_O_5_ nutrient weight, 619 kt of phosphate use was from DAP, with 103 kt from NP, and 16 kt from single superphosphate (SSP; Ca(H_2_PO_4_)_2_; 0:14–18:0). There is negligible use of phosphate from triple superphosphate (TSP; Ca(H_2_PO_4_)_2_. H2O; 0:46:0) or NPK sources. Similarly, there is minimal potash fertiliser usage of just 21 kt in 2012/13 (NFDC 2013). This included 8 kt of sulphate of potash (SOP; K_2_SO_4_; ~0:0:50), 3.6 kt of muriate of potash (MOP; KCl; ~0:0:60), and the rest as NPK fertilisers.

**Fig. 2:**
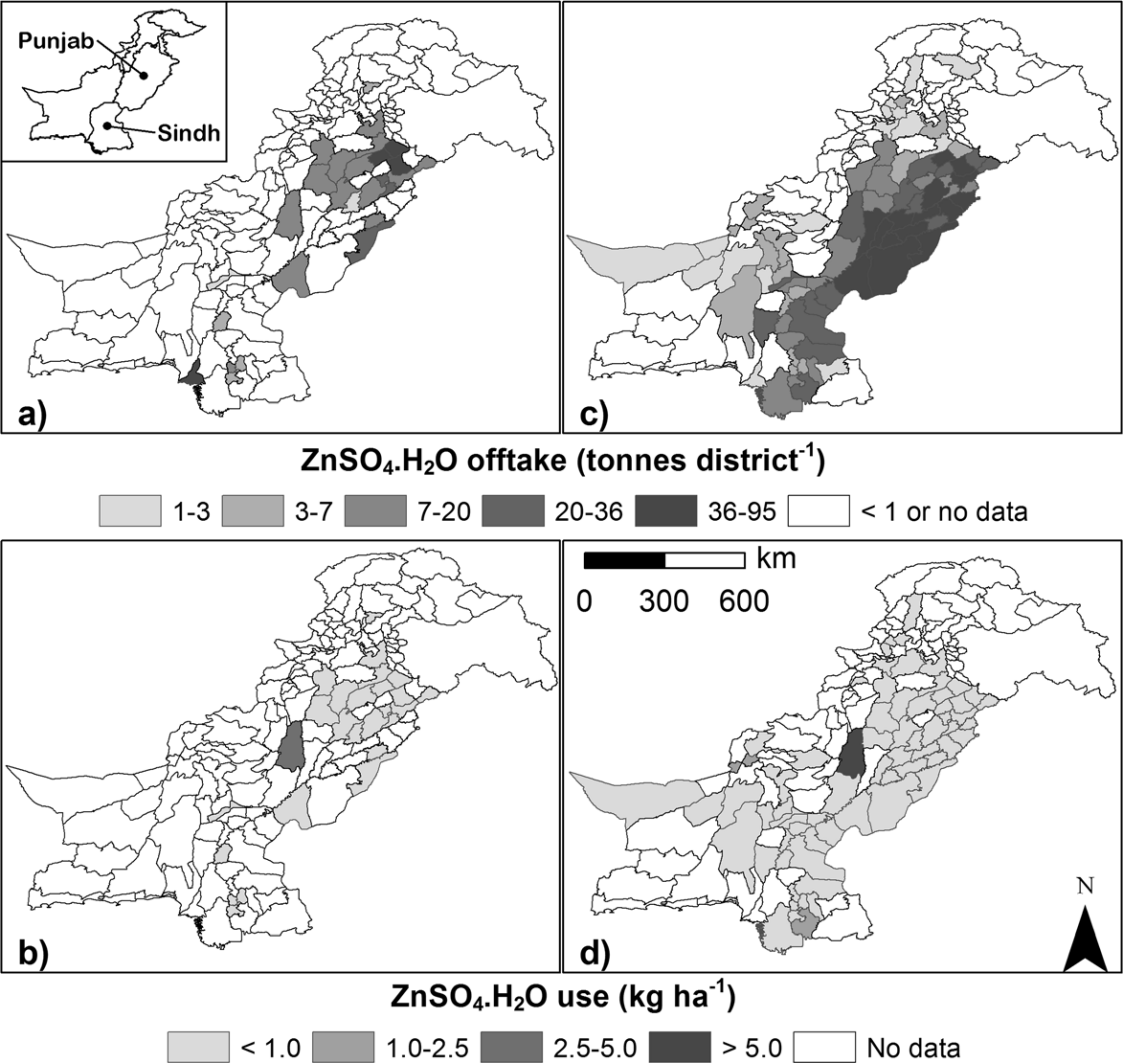
Zinc fertiliser use in Pakistan by District. **a**, **b**, data for Engro Fertilizer Ltd. for 2012/13 (NFDC 2013). **c**, **d**, unpublished data of Fauji Fertilizer Company Ltd., Rawalpindi, Pakistan (2014 calendar year). Units are tonnes of ZnSO4.H_2_O equivalents (**a**, **c**) and kg ha-^1^ based on wheat production area as a proxy for suitable land (**b**, **d**). Pakistan’s district shape files are from Global Administrative Areas (GADM) database (http://www.gadm.org/). Data represent >50 % of Zn market

Data for micronutrient fertiliser-use in Pakistan are not widely available, although usage is undoubtedly low. Baseline Zn fertiliser-use data for the 2012/13 cropping season were obtained from NFDC (2013), however, these include data solely for Engro Fertilizer Ltd. (Fig. 2a, b; NFDC, 2013). Data from Fauji Fertilizer Company Ltd. are from 2014 (Fig. 2c, d; Munir H. Zia, unpublished data, Fauji Fertilizer Company Ltd., FFC, Rawalpindi). Other major companies such as Fatima Fertilizer Company have recently started selling Zn fertilisers but sales data are not yet available. Fauji and Engro are likely to represent >50 % of the current Zn fertiliser market in Pakistan. It can be concluded from these data sources that the current use of Zn fertilisers in Pakistan is low (Fig. 2). Estimated annual use of Zn products on wheat is 7300 t (i.e. ZnSO_4_. H2O equivalents, 33 % Zn). Product use comprises 5100 t ZnSO_4_. H2O which are applied to soils in granular or powder forms, and 10,700 t of liquid Zn, including both ZnSO_4_.H_2_O and chelated Zn forms which is equivalent to 2200 t of granular ZnSO_4_. H2O. This represents a granular:foliar Zn-use ratio of ~0.7:0.3. Zinc fertiliser-use, i.e. usage divided by area under wheat production, is therefore <<1 kg ha^−1^ for most districts in Pakistan. Input costs for Zn were assumed to be US$ 1600 t^−1^ ZnSO_4_.H_2_O (2015 value; Munir H. Zia, personal communication). This amount is the approximate amount paid by the farmer, which comprises the cost of the raw product received at the port (~50 % of the cost in 2015), plus other costs including product unloading, transport, packaging, administration, and profit margin. Zinc fertilisers are used most widely in Punjab and Sindh, with limited use elsewhere in Pakistan (Fig. 2).

**Fig. 3:**
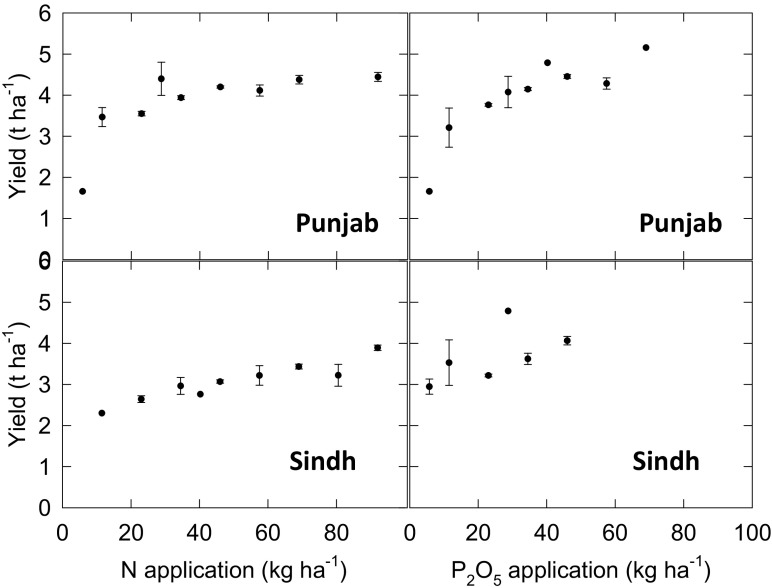
Yield-responses of wheat to N (urea) and P_2_O_5_ (di-ammonium phosphate, DAP) applications. Data are from 1193 and 1338 farmers in Punjab and Sindh Provinces, respectively, from a survey conducted in early 2015 from 2014 harvest data (Waqar Ahmad, personal communication, Food and Agriculture Organization of the United Nations, FAO, Islamabad)

Baseline data for on-farm Zn fertiliser-use were obtained for Punjab and Sindh from a survey of >2500 farmers taken in early 2015 (Waqar Ahmad, personal communication, Food and Agriculture Organization of the United Nations, FAO, Islamabad). As part of a broader study, farmers were asked to recall information including farm-size, major crop types and yields from the 2014 harvest (i.e. wheat, rice, cotton, sugarcane, maize, other). They were also asked to recall crop-specific fertiliser usage, in terms of urea and CAN use for N; DAP/MAP/SSP/NP use for P; MOP/SOP use for K; Zn and B use). In this way, data were obtained from 1193 and 1338 farmers in Punjab and Sindh Provinces, respectively. There were consistent positive relationships between N and P_2_O_5_ application rates and wheat yield (Fig. 3), confirming that these survey data are a useful proxy for this current study. For example, wheat yields increased from ~2 t ha^−1^ at the lowest N application rates, to 4.4 and 3.9 t ha^−1^ at an application rate of 92 kg N ha^−1^.

**Fig. 4:**
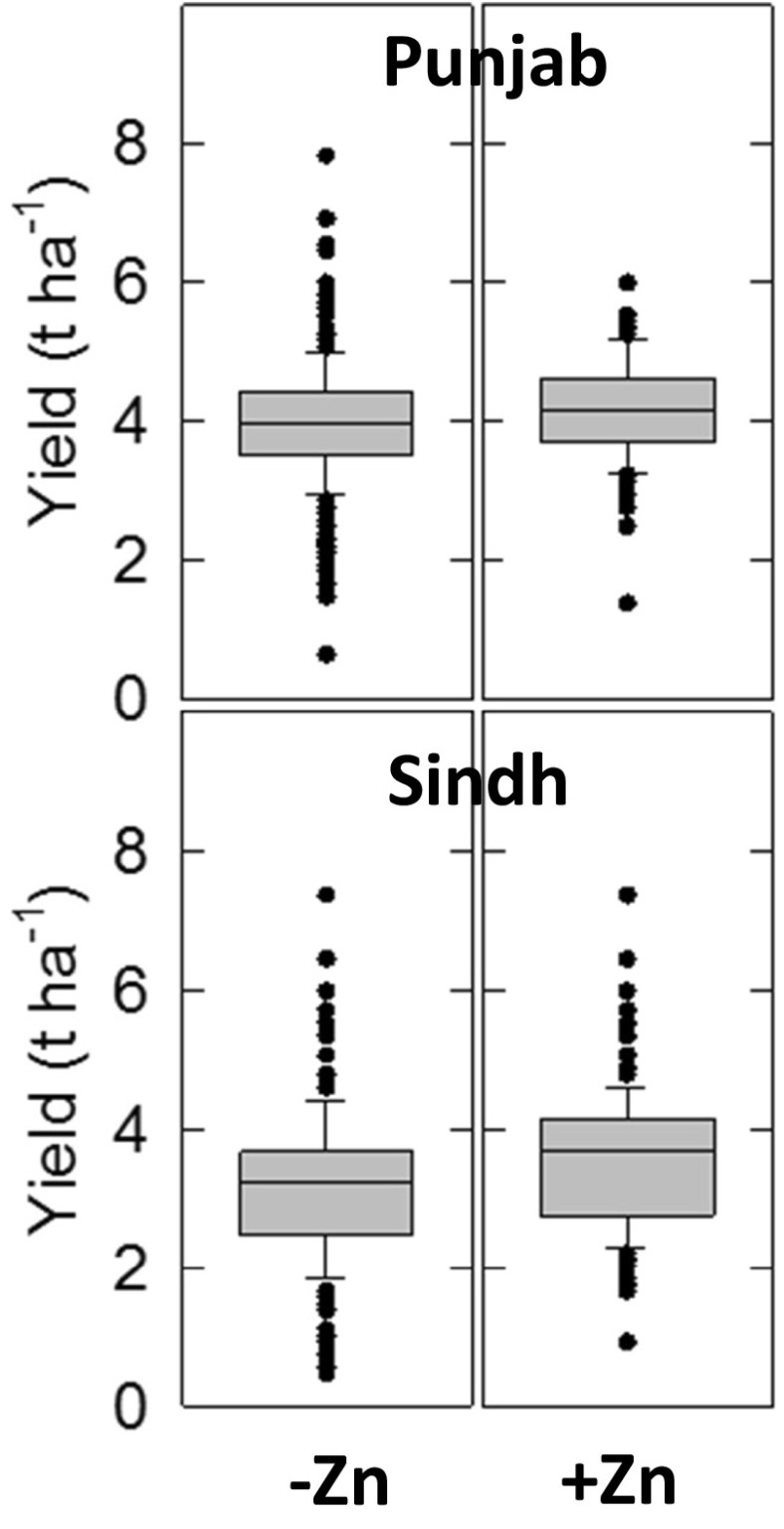
Yield-response of wheat to Zn applications. Data are from 1193 and 1338 farmers in Punjab and Sindh Provinces, respectively, from a survey conducted in early 2015 from 2014 harvest data (Waqar Ahmad, personal communication, Food and Agriculture Organization of the United Nations, FAO, Islamabad). In Punjab, 1021 and 172 farmers reported zero-Zn (−Zn) and Zn fertiliser use (+Zn), respectively. In Sindh, the equivalent figures were 1028 and 310. Boxes represent the middle two quartiles; whiskers represent the 95 % confidence limits; outliers circled. Median increases are indicated by the horizontal line within the box. Yield increases in both Punjab and Sindh were significant (*P* < 0.001)

In Punjab and Sindh, 14.4 and 23.2 % of farmers, respectively, reported using Zn fertilisers on wheat. This corresponds to a total area receiving Zn fertiliser of 1.1 and 0.4 Mha in Punjab and Sindh, respectively. Among farmers reporting Zn fertiliser-use, the mean application rate was 4.8 kg ZnSO_4_.H_2_O ha^−1^, based on dividing this area by 7.3 kt. The mean wheat yield without Zn fertilisers was 4.0 and 3.1 t ha^−1^ in Punjab and Sindh Provinces, respectively (Fig. 4).

### Wheat production data and support prices

Area under wheat production was derived from the Pakistan Agriculture Census, with 7.5 and 1.9 M ha in Punjab and Sindh provinces, respectively (PBS 2010; Table 1). The mean yield is 2.5 and 1.9 t ha^−1^ in Punjab and Sindh, respectively (PBS 2009), of which 90 % and 97 % is irrigated (PBS 2010). Output prices of wheat are based on a support price of US$ 312 t^−1^ (October 2015 value; Munir H. Zia, personal communication).

**Table 1:** Wheat yields and production for Punjab and Sindh Provinces, Pakistan. Data from Pakistan Bureau of Statistics (2010), which reports that 90 and 97 % of wheat is irrigated in Punjab and Sindh, respectively

	Total area cropped (Mha)	Proportion of cropped area in wheat (%)	Cropped area of wheat (Mha)	Wheat production (Mt)	Wheat mean yield (t ha-^1^)
Punjab	18.2	41	7.5	18.4	2.5
Sindh	5.0	38	1.9	3.5	1.9
Pakistan	~27.5	~37	~10	~25	~2.7

### Disease burden due to Zn deficiency

An estimated 323,214 DALYs were lost due to Zn deficiency in Pakistan in 2010 on the basis of dietary Zn deficiencies, the prevalence of stunting, and a total population of 182 M (Wessells and Brown 2012; Lim et al. 2012; IHME 2014; UNDP 2013). This is equivalent to 187 DALYs lost 100 k^−1^ population. Recent population data for Punjab and Sindh are not available in the public domain, however these provinces comprised 54 % and 22 % of national population in 1998 (PCO 1998) so their 2010 populations were estimated to be 92.9 M and 38.4 M, respectively. Thus, in 2010, 173,506 and 71,746 DALYs were lost *pro rata* *per annum* in Punjab and Sindh, respectively. As dietary Zn supplies increase, we assume that DALYs lost due to Zn deficiency will decrease at the same rate as the national prevalence of Zn deficiency from a baseline of 24.3 % and 22.8 % in Punjab and Sindh, respectively, and assuming no additional food consumption (Supplementary Tables 3, 4).

### Assumptions used to simulate increased Zn fertiliser-use

To estimate the value of potential increased Zn fertiliser usage, an instantaneous, area-based approach was adopted, with no time dimension or discounting factors. This is justified because Zn fertilisers could be deployed immediately by incorporating Zn with existing granular N fertilisers or pesticide/fungicide sprays. We assumed that the proportion of wheat fertilised at 4.8 kg ha^−1^ could incrementally increase from the current 14.4 and 23.2 % of the areas currently under wheat production in Punjab and Sindh, respectively, up to 100 % of these areas. The granular:foliar Zn fertiliser-use ratio of ~0.7:0.3, based on current industry estimates, was assumed to remain constant.

**Fig. 5:**
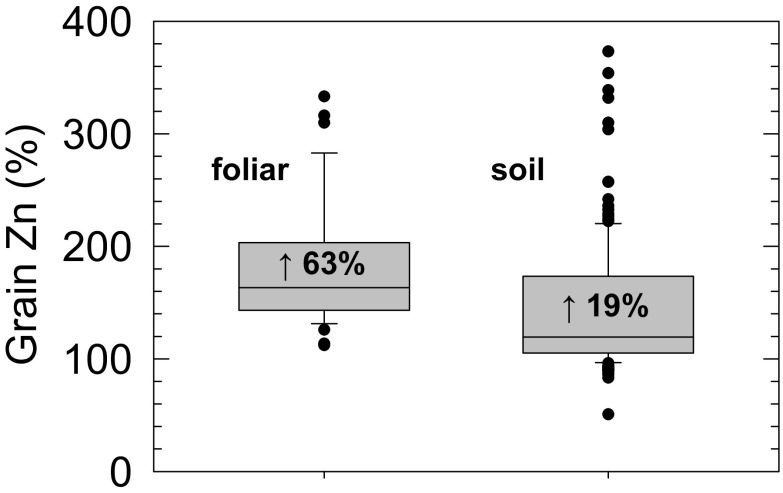
Increases in wheat grain Zn concentration as a result of foliar- or soil-applied/granular Zn fertilisers compared to unfertilised controls. Boxes represent the middle two quartiles; whiskers represent the 95 % confidence limits; outliers circled. Median increases are indicated by the horizontal line and text within the box. Data are from a literature-survey of Joy et al. (2015b)

Grain yields were assumed to increase by 8 % and 14 % under Zn fertilisation in Punjab and Sindh, respectively, according to median data from the farmer survey (Fig. 4). These increases were both highly significant (Student's t-tests; *P* < 0.001). This represents an area-normalised potential yield increase of 9 % for Punjab and Sindh combined. These survey data are therefore consistent with median yield increases of 16 % following soil Zn application in six site-years in Pakistan (Zou et al. 2012) and 10–11 % from applications of Zn fertilisers to maize, rice and wheat in a recent global literature review (Joy et al. 2015b). The potential increases in grain Zn concentration in wheat resulting from granular and foliar Zn fertiliser-use were taken from this same review. From 15 studies of wheat, comprising 196 combinations of location, cultivar and application rate, granular (soil-applied) and foliar Zn fertilisers resulted in median increases in grain Zn concentration of 19 % and 63 %, respectively (Fig. 5; Joy et al. 2015b). We therefore assumed that granular fertilisers increased grain Zn concentration by 19 %, from a baseline of 23.6 mg kg^−1^ (i.e. the 24.9 mg kg^−1^ was adjusted to assume zero Zn fertilisation) to 28.1 mg kg^−1^, and that foliar fertilisers increased grain Zn concentration by 63 % to 38.5 mg kg^−1^. Thus, the weighted mean increase in grain Zn concentration under Zn fertilisation was assumed to be 32.2 %, giving a concentration of Zn-fertilised wheat grain of 31.2 mg kg^−1^. All calculations are presented in Supplementary Tables 2–4.

The value of one DALY was monetised using multiples of Gross National Income (GNI) per capita, converted to International Dollars (I$) based on purchasing power parity (GNI_PPP_). An I$ has the equivalent purchasing power as a US$ dollar in the US based on the 2011 International Comparison Program. A per capita GNI_PPP_ in Pakistan of I$ 5110 (http://data.worldbank.org/indicator/NY.GNP.PCAP.PP.CD) was used based on data in October 2015.

## Results

### Potential increases in dietary Zn supply with increased Zn fertiliser-use

**Fig. 6:**
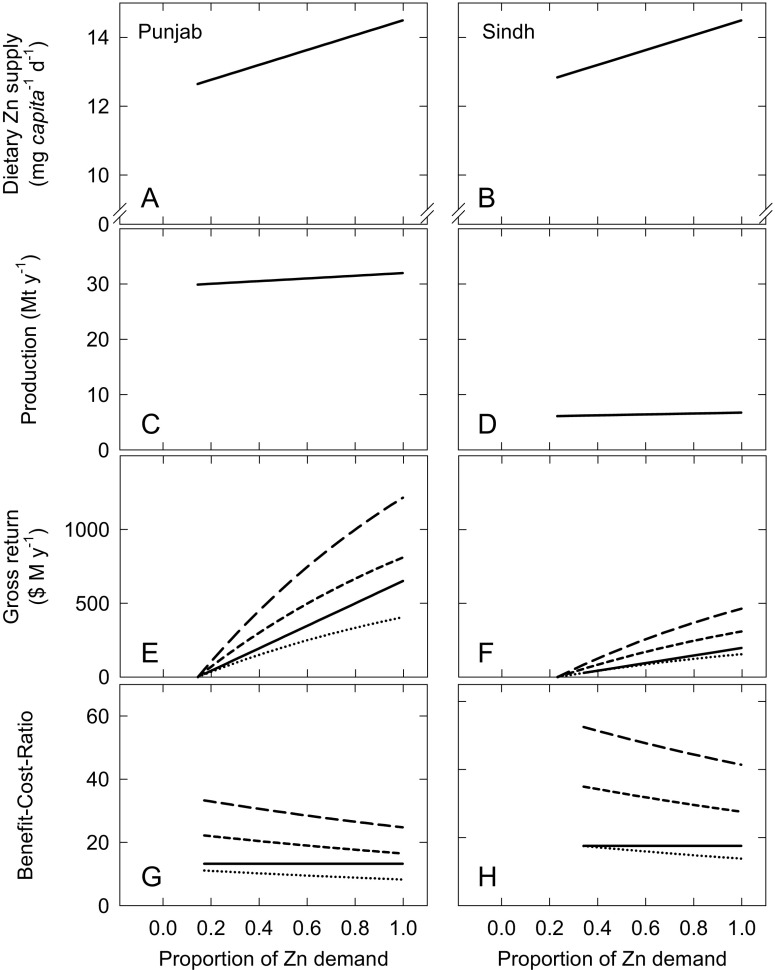
Projected responses to increasing Zn fertiliser use in Punjab (**a**, **c**, **e**, **g**) and Sindh (**b**, **d**, **f**, **h**) Provinces, Pakistan. (**a**, **b**) Dietary Zn supply from all food sources; (**c**, **d**) total wheat yield. (**e**, **f**) Gross financial return in which yield (*solid line*) is expressed in current US Dollars (US$) and health benefits are expressed in International Dollars (I$) in which the value of a Disability Adjusted Life Year (DALY) is expressed as a 1 (*dotted line*), 2 (*short-dashed line*) or 3 (*long-dashed line*) multiples of Gross National Income based on parity purchasing power (GNIPPP) which is US$ 5110 for Pakistan from the 2011 International Comparison Program. (**g**, **h**) Benefit-Cost-Ratios for Zn use on wheat based on the ratio of return per unit input cost; lines labelled as described previously

If all wheat was fertilised with Zn, we project that dietary Zn supply would increase from 12.6 and 12.8 mg *capita* ^−1^ d^−1^ in Punjab and Sindh, respectively, to 14.5 mg *capita* ^−1^ d^−1^ (Fig. 6a, b). Zinc deficiency prevalence would decrease from 24.8 and 23.1 %, to 12.9 and 12.7 %, in Punjab and Sindh, respectively.

### Potential increases in wheat production with increased Zn fertiliser-use

A projected increase in wheat yield in Punjab from 4.0 to 4.3 t ha^−1^ would translate to an overall increase in production from 29.9 to 32.0 Mt y^−1^ (Fig. 6c) if the entire current area under wheat production was fertilised with Zn. This represents a potential increase in the monetary value of Punjab’s wheat harvest from US$ 9300 M to US$ 10,000 M (Fig. 6e). Expenditure on Zn fertilisers would increase by US$ 49 M, at a BCR of 13.3 (Fig. 6g). An increase in wheat yield in Sindh from 3.2 to 3.6 t ha^−1^ would translate to an overall increase in production from 6.1 to 6.7 Mt y^−1^ (Fig. 6d). This represents a potential monetary increase in the value of Sindh’s wheat harvest from US$ 1900 M to US$ 2100 M (Fig. 6f). Expenditure on Zn fertilisers would increase by US$ 11.2 M, which equates to a BCR of 17.5 (Fig. 6h). These BCRs are based on 2015 prices of US$ 1600 t^−1^ for ZnSO_4_.H_2_O and a wheat support price of US$ 312 t^−1^. These BCRs are greater than those reported previously, which ranged from 1.3:1 to 11:1, with a mean of 7:1 (NFDC 1998). However, at the end of 1997, the price to farmers of ZnSO_4_.H_2_O was US$ 2378 t^−1^ and the wheat support price was US$ 136 t^−1^ (Munir H. Zia, personal communication). Our current BCR estimates are therefore expected to be >3-fold greater than for estimates based on the equivalent inputs and yields in 1997, and our data are therefore consistent with previous studies in the region (NFDC 1998). It is important to note that our baseline estimates of wheat production for each province are extrapolated from grain yields reported in the farmer survey, which are greater than actual yield averages for Pakistan. Thus, our baseline estimates of wheat production exceed actual current wheat production.

### Decreases in DALYs lost

Given the projected increase in dietary Zn supply, the number of DALYs lost annually due to Zn deficiency would decrease from 174 k to 94 k in Punjab, and from 72 k to 42 k in Sindh. Valuing one DALY at the average national *per capita* income, these reductions in disease burden represent a gross return of up to I$ 405 M and I$ 155 M in Punjab and Sindh, respectively, based on a GNI_PPP_ of I$ 5110 *capita* ^−1^ in Pakistan (Fig. 6e, f). These gross returns would multiply if a monetary value of double or triple *per capita* income were used, as suggested by the WHO, and these data are presented alongside for comparison. In terms of cost *per* DALY saved, these range from I$ 461 to I$ 619 and I$ 292 to I$ 370 in Punjab and Sindh, respectively. As such, the cost *per* DALY saved is well below the average *per capita* income in Pakistan and therefore Zn fertilisation in Punjab and Sindh can be considered a cost-effective public health intervention by WHO standards (WHO 2001).

The cost *per* DALY saved increases slightly as Zn fertiliser-use increases. In Punjab, the BCR of Zn fertiliser-use therefore decreases from 11.1 to 8.3 when one DALY is valued at the average national *per capita* income (Fig. 6g). The equivalent figures for Sindh show a decrease from 17.5 to 13.8 (Fig. 6h). This decrease is due to the use of the Estimated Average Requirement (EAR) cut-point method to define the proportion of the population that is at risk of dietary Zn deficiency (Kumssa et al. 2015b). Because a normal distribution of Zn intakes is assumed for the population, when the mean population intake is >EAR, an increasingly smaller proportion of the population is moved out of deficiency for each projected incremental increase in Zn intake. In addition to increasing dietary Zn supply, the annual increase in grain supply would represent 19 kg *capita* ^−1^ across Punjab and Sindh which would improve general food security in terms of energy supply. This additional potential benefit is not factored into the current analysis.

## Discussion

Our analysis shows there are likely to be substantial financial returns from an increase in Zn fertiliser-use in Pakistan, in terms of both yield and public health benefits. Our analysis is likely to underestimate these returns, based on the input data used, for several reasons. First, the current extent of dietary Zn deficiency in Pakistan is likely to be much greater than our assumed baseline of 23.9 % according to the 2011 Pakistan National Nutrition Survey Report (NNS 2011). Second, no account has been taken of residual benefits of Zn fertilisers on subsequent crop yields or grain Zn composition. However, residual effects are likely to provide benefits for several years for both wheat and other crops (NFDC 1998; Singh and Shivay 2013; Manzeke et al. 2014; Wang et al. 2015b). Third, foliar Zn applications could make up a larger proportion of future Zn fertiliser-use, especially if it is applied in conjunction with pesticide sprays to minimise additional costs of application (Wang et al. 2015a; Ram et al. 2016). Fourth, if other hostile soil factors, such as B deficiency or salinity/sodicity, are addressed through soil management practices to improve yields, then greater returns *per* unit input of Zn are possible (NFDC 1998). Fifth, with yield increases, there is likely to be greater food consumption *per se* which is not accounted for in this study, although yield increases are likely to be minimal under foliar applications of Zn fertilisers (Joy et al. 2015b). Of course, if there is an increased supply of wheat, then this could reduce prices, and therefore reduce farmers’ financial return from higher yields. However, greater yields would also likely increase the consumption of wheat both directly and indirectly *via* livestock. Thus both Zn and energy intake would likely increase, especially of low-income consumers, and therefore improve national food security. Given the commodity status of wheat and Pakistan’s integration into global markets, an increase in wheat yields is not expected to have an impact on local prices.

To our knowledge, this is the first study that has combined both yield and health-based valuations to quantify the benefits of an increased projected supply of micronutrients through fertilisers. The potential BCRs relating to reductions in DALYs lost each year – based on valuing DALYs conservatively at the national per capita income – are similar in scale to BCRs based on increased yield outputs based on increased Zn fertiliser-use. However, if multiples of the *per capita* income are used to estimate BCRs for reducing the number of DALYs lost each year, then the potential public health benefits of projected increases in Zn fertiliser-use far exceed returns that result from increased yields.

Such estimates of BCRs have profound implications regarding the question ‘who pays?’ for such a programme. There is clearly scope for farmers to pay for Zn fertilisers based on yield-related BCRs. However, this requires trust in the quality of fertilisers, access to fertilisers, commodity markets and credit, and adequate extension services. Asking consumers to pay a premium for wheat grain with increased concentrations of Zn is perhaps more challenging. A recent survey on the potential of iodine biofortification strategies in Uganda showed that despite some awareness among parents and school authorities of the essential role of adequate iodine for health, and salt iodisation programmes, there was little awareness of the potential role iodine biofortified foods could play (De Steur et al. 2015). However, consumers’ willingness-to-pay for iodine biofortified vegetables is likely to increase in parallel with awareness of their potential health benefits (Mogendi et al. 2015).

Given the potential impact of Zn fertilisers on public health at the macroeconomic scale, including reduced health care costs and improved productivity, there seems to be a strong case for governments to consider subsidy programmes for Zn fertilisers. Furthermore, there may be additional micronutrient deficiencies that could be addressed through agronomic approaches, e.g. as discussed by Chilimba et al. (2012) and Joy et al. (2015a) for Malawi. In such cases government interventions could help overcome the lack of incentive that farmers have to pay higher prices for fertilisers that are also enriched with selenium or iodine, given that these minerals lack yield benefits. Agronomic approaches to deliver micronutrients also have the potential to reach wider sections of the community, including those who are socially excluded from fortification programmes which are delivered in schools or other community settings, for example, children with disabilities (Kuper et al. 2015).

While our framework to value fertiliser-based approaches to alleviating dietary mineral deficiencies provides a strong initial argument for pursuing such approaches further, based on economic and cost-effectiveness considerations, we acknowledge that attention should be paid to ensure that: (1) the increased Zn supply through cereals is sufficiently bioavailable to have a dietary impact (e.g. Fredlund et al. 2006); (2) EARs are not over-estimated for the target population; (3) toxicity risks can be excluded; (4) the increased Zn supply does not exacerbate other micronutrient deficiencies or diseases; (5) decision-makers evaluate available options to improve public health and nutrition on a case-by-case basis to optimise the policy mix both in terms of targeting and budgetary considerations; (6) sufficient attention is paid to generate and use accurate baseline data, at sufficiently high geospatial resolution, of the nutritional composition of soils, crops and food, and of the nutritional needs of people.

Given the prevalence of dietary Zn deficiency globally, its alleviation falls clearly under the second of the United Nation’s Sustainable Development Goals (UN 2015), which calls for an end to hunger, including access to sufficiently nutritious food by 2030. We hope that this case-study provides a useful quantitative framework for assessing the potential impact of increased Zn supply in food systems from agronomic and breeding approaches to increase the Zn composition of food crops.

## Electronic Supplementary Material

ESM 1 (XLSX 49 kb)
